# Cartilage–Specific Over-Expression of CCN Family Member 2/Connective Tissue Growth Factor (CCN2/CTGF) Stimulates Insulin-Like Growth Factor Expression and Bone Growth

**DOI:** 10.1371/journal.pone.0059226

**Published:** 2013-03-28

**Authors:** Nao Tomita, Takako Hattori, Shinsuke Itoh, Eriko Aoyama, Mayumi Yao, Takashi Yamashiro, Masaharu Takigawa

**Affiliations:** 1 Department of Biochemistry and Molecular Dentistry, Okayama University Dental School, Okayama, Japan; 2 Department of Orthodontics, Okayama University Graduate School of Medicine, Dentistry, and Pharmaceutical Sciences, Okayama University Dental School, Okayama, Japan; 3 Biodental Research Center, Okayama University Dental School, Okayama, Japan; University of Western Ontario, Canada

## Abstract

Previously we showed that CCN family member 2/connective tissue growth factor (CCN2) promotes the proliferation, differentiation, and maturation of growth cartilage cells *in vitro*. To elucidate the specific role and molecular mechanism of CCN2 in cartilage development *in vivo*, in the present study we generated transgenic mice overexpressing CCN2 and analyzed them with respect to cartilage and bone development. Transgenic mice were generated expressing a *ccn2/lacZ* fusion gene in cartilage under the control of the 6 kb-*Col2a1*-enhancer/promoter. Changes in cartilage and bone development were analyzed histologically and immunohistologically and also by micro CT. Primary chondrocytes as well as limb bud mesenchymal cells were cultured and analyzed for changes in expression of cartilage–related genes, and non-transgenic chondrocytes were treated in culture with recombinant CCN2. Newborn transgenic mice showed extended length of their long bones, increased content of proteoglycans and collagen II accumulation. Micro-CT analysis of transgenic bones indicated increases in bone thickness and mineral density. Chondrocyte proliferation was enhanced in the transgenic cartilage. In *in vitro* short-term cultures of transgenic chondrocytes, the expression of *col2a1, aggrecan* and *ccn2 genes* was substantially enhanced; and in long-term cultures the expression levels of these genes were further enhanced. Also, *in vitro* chondrogenesis was strongly enhanced. *IGF-I* and *IGF-II* mRNA levels were elevated in transgenic chondrocytes, and treatment of non-transgenic chondrocytes with recombinant CCN2 stimulated the expression of these mRNA. The addition of CCN2 to non-transgenic chondrocytes induced the phosphorylation of IGFR, and *ccn2*-overexpressing chondrocytes showed enhanced phosphorylation of IGFR. Our data indicates that the observed effects of CCN2 may be mediated in part by CCN2-induced overexpression of IGF-I and IGF-II. These findings indicate that CCN2-overexpression in transgenic mice accelerated the endochondral ossification processes, resulting in increased length of their long bones. Our results also indicate the possible involvement of locally enhanced IGF-I or IGF-II in this extended bone growth.

## Introduction

CCN2(CCN family 2)/CTGF (connective tissue growth factor) is a member of the CCN family of secreted proteins, which also includes Cyr61/CCN1, NOV/CCN3, WISP1/CCN4, WISP2/CCN5, and WISP3/CCN6. CCN2 regulates diverse cell functions including mitosis, adhesion, apoptosis, extracellular matrix (ECM) production, growth arrest, and cellular migration [1], [2]. The multimodular character of CCN factors allows multiple interactions between them and other growth factors such as TGFß, BMPs, IGFs or VEGF and networking between growth factors, extracellular matrix, and cell-surface receptors such as integrins [3]. Thus, it is not surprising that CCN factors are involved in a multiplicity of effects during development, differentiation, wound healing, and disease states, including tumorigenesis and fibrosis [2]. Most prominently, CCN2 has emerged as a major regulator of chondrogenesis, angiogenesis, and fibrogenesis [4]. CCN2 induces the migration of endothelial cells [5], [6], [7] and stimulates the synthesis of matrix proteins including collagens and fibronectin [8], [9]. It is expressed in various tissues, with highest levels found in prehypertrophic chondrocytes and vascular tissues in developing embryos (for reviews, see refs [4], [10]. Previously we demonstrated in a series of *in vitro* studies that CCN2 stimulates both the proliferation and synthesis of type II collagen and proteoglycans of growth-plate chondrocytes [11], human chondrosarcoma-derived chondrocytic cells [11], [12], articular chondrocytes [13], and auricular chondrocytes [14]. Moreover, it induces hypertrophy and calcification of growth-plate chondrocytes, but not those of articular or auricular chondrocytes [11], [14], [15]. Also, osteoblast proliferation and maturation are stimulated by CCN2 [16]. These *in vitro* findings are consistent with studies on CCN2-deficient mice, which develop skeletal dysmorphisms including kinky bone and cartilage elements, due to impairment of chondrocyte proliferation and extracellular matrix deposition in the hypertrophic zone [17]. As a result of CCN2 deficiency, growth-plate angiogenesis and endochondral ossification are partially impaired, and CCN2-deficient mice die after birth because of respiratory failure caused by the skeletal defects [17]. Although multiple effects of CCN2 on differentiation, proliferation, and matrix synthesis of chondrocytes, fibroblasts, endothelial cells, and osteoblasts have been reported, the specific role of CCN2 synthesized by chondrocytes during cartilage and bone development *in vivo* remains unclear.

To elucidate the role of chondrocyte-derived CCN2, we generated CCN2-over-expressing mice with the gene expressed under the control of a 6 kb-*Col2a1* promoter that included a cartilage-specific enhancer element in the first intron of the *Col2a1* gene and obtained *in vivo* evidence for a key role of CCN2 in regulating chondrocyte gene expression and cartilage differentiation. Furthermore, our data suggest that CCN2 regulates the endochondral ossification process in long bones partially through increased expression of IGF-I and IGF-II.

## Materials and Methods

### Generation of Transgenic Mice

**Figure 1:**
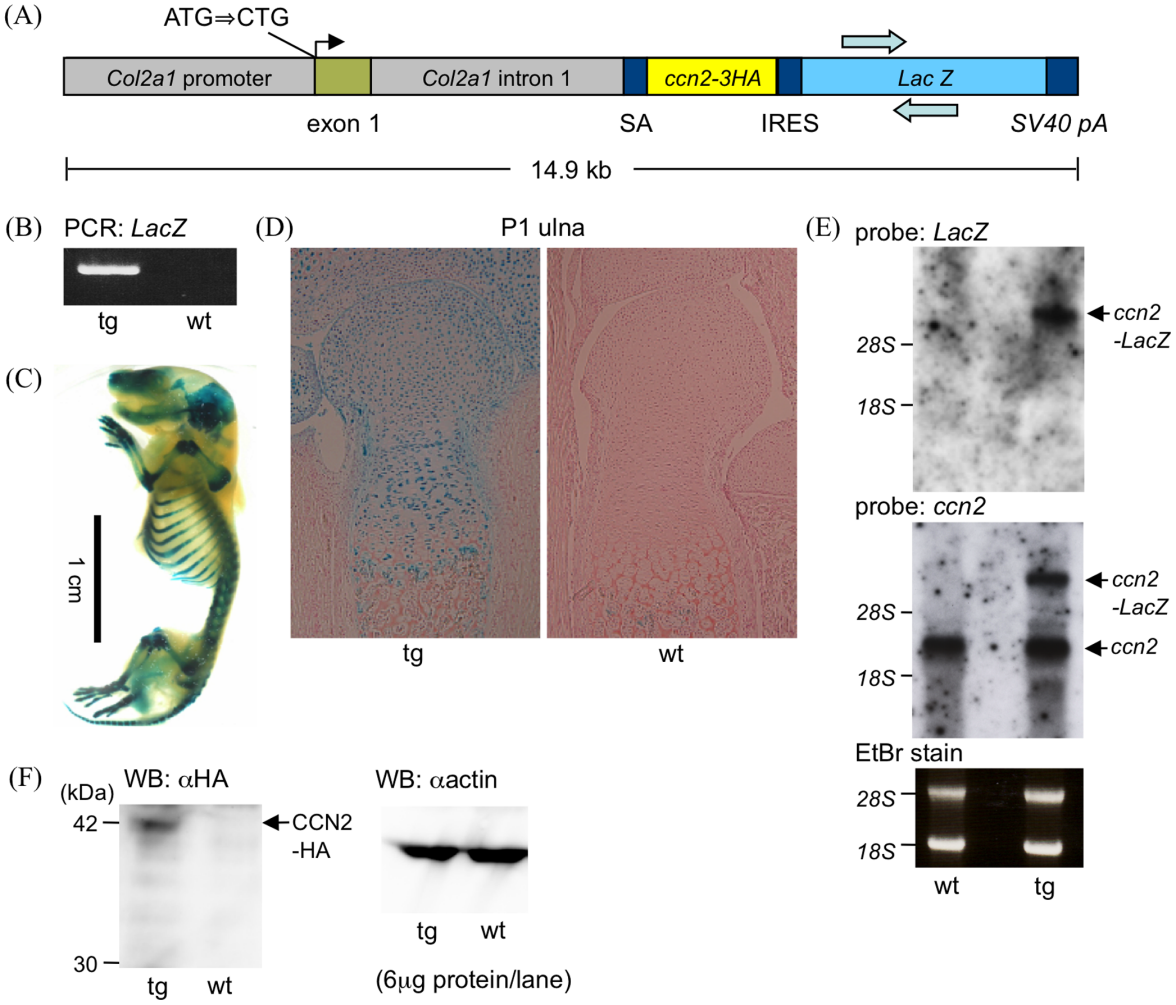
Generation of *Col2a1-ccn2* transgenic mice. (A) Schematic representation of the construct of the expression of HA-tagged CCN2 and IRES-LacZ in chondrocytes driven by the 6-kb *Col2a1* promoter-enhancer. The original initiation codon of *Col2a1* was mutated to CTG to facilitate translation from downstream cDNA. (B) Genotyping of transgenic mice (tg) by PCR to detect the transgene. wt, wild type. The location of the primers used are indicated in “A” by arrows. (C) Skeletal preparation of a newborn mouse after whole-mount X-gal staining, showing cartilage-specific expression of the transgene. (D) Sagittal sections of ulnae from wt and tg after whole-mount X-gal staining. All of the cartilaginous cells showed X-gal staining. The sections were counter-stained with Safranin-O. (E) Analysis of transgene expression by Northern hybridization using total RNA from tg and wt cartilage. *LacZ* (top) and *ccn2* (middle) probes were used to detect transgenic and endogenous *ccn2*, respectively. (F) Western blot (WB) analysis using cell lysates from tg and wt cartilage and anti-HA antibody recognizing only the CCN2-HA transgene products (left blot). The HA-tagged CCN2 was expressed in cartilage of tg mice. A Western blot of the same cell lysate reacted with anti-actin antibody as a loading control is also shown (right blot).

To express the *ccn2* as transgene in chondrocytes, we cloned the cDNA encoding a HA-tagged mouse *ccn2* gene into a vector containing 3 kb of the *Col2a1* promoter and 3.02 kb of the intron 1 sequence [18], [19]. The *LacZ* gene preceded by an internal ribosomal entry site was placed downstream of the *ccn2* cDNA (Fig. 1A). This construct was microinjected into the pronuclei of fertilized C57BL/6CrSlc eggs to generate transgenic mice. Routine genotyping to identify the transgene was done by detecting the *LacZ* gene by performing a polymerase chain reaction (PCR) on genomic DNA. The primer sequences used were 5-GCATCGAGCTGGGTAATAAGCGTTGGCAAT-3′ and 5-GACACCAGACCAACTGGTAATGGTAGCGAC-3′.

All experimental procedures were performed in accordance with the Guidelines for Proper Conduct of Animal Experiments of the Science Council of Japan and approved by the Animal Research Control Committee of Okayama University (Approval No.: OKU-2012113).

### LacZ Staining and Skeletal Preparation

LacZ activity was detected by staining with X-gal (5-bromo-4-chloro-3-indolyl-D-galactopyranoside; Roche) for 3–6 hours following fixation with glutaraldehyde and formaldehyde as described earlier [20]. For staining of embryos older than 15.5 days, the skin and internal organs were removed before fixation. LacZ-stained embryos were postfixed overnight in 4% formaldehyde, dehydrated, and embedded in paraffin. Sections were counterstained with eosin. Some LacZ-stained embryos were cleared with KOH –glycerol. Skeletal morphology was analyzed by alizarin red and alcian blue staining followed by clearing with 1% (w/v) KOH [21], [22].

### RNA Preparation and Northern Hybridization

RNA was prepared either directly from cartilage or from chondrocyte cultures. For the direct RNA preparation, rib cages of E18.5 or 19.5 embryos were separated from soft tissues, and single ribs were isolated. The isolated ribs were separated from bone, and the cartilage was soaked in Isogen (Nippon Gene) and homogenized until the tissue clumps had disappeared. The cartilage RNA were purified according to the Isogen instructions, and the purified RNA were further cleaned by using the RNeasy kit (Qiagen). For the RNA preparation from chondrocytes, the cells from rib cartilage were cultured as described below, harvested, and then subjected to RNA purification using the RNeasy kit. For Northern hybridization, 10 µg of RNA from costal cartilage was resolved on an agarose gel, transferred onto a nylon membrane (Bio-Rad), and hybridized with [^32^P]-labeled *LacZ* or *ccn2* probes as described previously [23].

### Western Blotting

Rib cartilage from E18.5 embryos was isolated as described above and homogenized with lysis buffer (50 mM Tris-HCl, pH 7.4, containing 150 mM NaCl, 1% Triton X-100, 0.1% SDS, and 1 mM PMSF). After centrifugation, the supernatant was collected; and 6 µg of protein per lane was loaded onto an SDS-PAGE gel. Western blotting was done as described previously [24] by using anti-HA (Covance), anti-actin (Sigma), anti-phospho IGF-1 receptor (Cell Signaling), and anti-IGF-1 receptor (Cell Signaling) antibodies.

### Histological Examination

For histological analysis, tissues from E17.5 and E19.5 embryos and from 1- and 3- day postnatal mice were fixed with 10% formaldehyde/PBS, demineralized with 0.5 M EDTA, and embedded in paraffin. Then 7 µm-thick-sections were stained with hematoxylin, eosin, and safranin-O. Immunohistochemical staining was performed by using a peroxidase-conjugated polymer (Nichirei, Japan) and anti-type II collagen MoAb (CII D3, [25] or anti-type X MoAb (X53, kindly provided by Dr. K. von der Mark, Germany, [26], [27]. For cell proliferation analysis, a PCNA staining kit (Zymed) was used. For detection of apoptotic cells, TUNEL analysis was performed by using an *In Situ* Cell Death Detection Kit, POD (Roche).

### Cell Cultures

For preparation of primary cultures, chondrocytes were isolated from the rib cages of 18.5- or 19.5-day embryos and/or newborn mice as described previously [28]. Briefly, the rib cages were digested with collagenase (0.1% collagenase P, Roche, in F12/DMEM containing 10% fetal calf serum) after adhering connective tissue and muscle had been thoroughly removed by trypsin pretreatment. The cells were grown to confluence for 1 month to hypertrophy in α-modification of minimum essential medium (α-MEM) containing 10% fetal bovine serum (FBS) and supplemented with 50 µg/ml of ascorbic acid with or without recombinant CCN2, and then harvested for RNA extraction.

For preparation of CCN2 recombinant protein, human *ccn2* cDNA was amplified by PCR and subcloned into the pET-15b vector (Novagen), which harbors a His-tag; and *E. coli* BL21(DE3)pLysS Rosetta strain cells were subsequently transformed with this vector. Expressed His-tagged CCN2 protein was purified by the use of Ni-NTA agarose.

For inhibition of autophosphorylation of IGF-1 receptor, the IGF-1R inhibitor PPP (Calbiochem) was used, at a concentration of 60 nM. Anti-CCN2 monoclonal antibody (11H3, kindly provided by Dr. Seto, Nippn Flour Mills Co., LTD.), which had an inhibitory effect on the CCN2-mediated enhancement of aggrecan gene expression was also used to inhibit this autophosphorylation.

### Quantitative real-time PCR

Reverse transcription (RT) was performed with 0.5 µg of total RNA as described above, and the resulting cDNA was amplified in triplicate by using the SYBR-Green PCR assay (TOYOBO SYBR Green PCR Master Mix; TOYOBO, Osaka, Japan), after which the products were detected with a LightCycler™ system (Roche, Basel, Switzerland). PCR reaction mixtures were incubated for 15 min at 95°C, followed by 50 amplification cycles of 30 s annealing at 60°C, 40 s extension at 72°C, and 30 s denaturation at 95°C. GAPDH was used to standardize the total amount of cDNA, as described previously [29].

The primers designed for real-time PCR were the following: *ccn2* (forward, 5'-GGTAAGGTCCGATTCCTACCAGG-3'; reverse, 5'-CTAGAAAGGTGCAAACATGTAAC-3'); *gapdh* (forward, 5′-GCCAAAAGGGTCATCATCTC-3′; reverse, 5′-GTCTTCTGGGTGGCAGTGAT-3′); *aggrecan* (forward, 5′-TCTTCAGTCCCGTTCTCCAC-3′; reverse, 5′-AACATCACTGAGGGCGAAGC-3′); *Col2a1* (forward, 5′-ATGACAA TCTGGCTCCCAACACTGC-3′; reverse, 5′-GACCGGCCCTATGTCCACACCGAAT-3′); *Col10a1* (forward, 5′-CCCAGGGTTACCAGGACAAA-3′; reverse, 5′-GTTCACCTCTTGGACCTGCC-3′); *vegf* (forward, 5′-CCCATGAAGTGATCAAGTTC-3′; reverse, 5′-ACCCGCATGATCTGCATGG-3′); *mmp9* (forward, 5′-GGAACTCACACGACATCTTCCA-3′; reverse, 5′-GAAACTCACACGCCAGAAGAATTT-3′); *IGF-I* (forward, 5′- GTGTGGACCGAGGGGCTTTTACTTC-3′; reverse, 5′-GCTTCAGTGGGGCACAGTACATCTC-3′); and *IGF-II* (forward, 5′-GTGGCATCGTGGAAGAGTGC-3′; reverse, 5′-GGGGTGGGTAAGGAGAAACC-3′); lacZ (forward, 5′-GGTTACGATGCGCCCATCTA-3′; reverse, 5′-ACGGCGGATTGACCGTAAT-3′).

### Micromass Culture

For preparation of micromass cultures, limbs from E11.5 embryos were digested in 0.05% trypsin for 1 hour on ice. After the cells had been suspended by pipeting, they were concentrated in 10% FCS-containing DMEM/F12 to 1×10^7^ cells/ml. Ten microliters of cell suspension containing 1×10^5^ cells was placed in the center of each well of a 24-well plate; and the cells were allowed to adhere to the bottom of the well for 1 h after the plate had been placed in an incubator (5% CO_2_, 37°C). Thereafter, 1 ml of culture medium was added to each well; and the medium was replaced every 24 hours. Cell condensation in the cultures was visible after 1 or 2 days, and cartilage nodules appeared after 3 days. Some cells were stained with Alcian blue (pH 1) to visualize cartilage, and others were harvested for extraction of total RNA.

### Analysis of Bone Mineralization

The femora from 8-week–old mice were removed, and the bones were scanned over the region from 1.2 mm to 4.0 mm from the distal epiphysial end by peripheral quantitative computed tomography (pQCT) analysis (XCT Research SA+[Stratec Medizintechnik GmbH, Pforzheim, Germany]). For the microcomputed tomography (micro-CT) analysis, the same position was scanned by using a Skyscan 1072 micro-CT machine (Skyscan, Aartselaar, Belgium).

## Results

### Cartilage-specific Over-expression of *ccn2* in Chondrocytes of Transgenic Mice Caused Increased Bone Size

For generation of transgenic mice over-expressing CCN2 in cartilage, HA-tagged *ccn2* cDNA was cloned into a vector containing 3 kb of the *Col2a1* promoter, 3.02 kb of the *Col2a1* intron 1 sequence, and *IRES-LacZ* (Fig. 1A). The purified vector DNA was injected into oocytes, and 2 founders tested positive for the *ccn2–lacZ* transgene by PCR (Fig. 1B) and were kept to establish transgenic lines. X-gal staining of newborn transgenic mice showed intense, cartilage-specific lacZ expression in all cartilage elements (Fig. 1C). In tissue sections of newborns, all growth-plate and resting chondrocytes were positive after X-gal staining, indicating that the expression domains of the transgene overlapped with those of endogenous *ccn2* (Fig. 1D; and see also [30]).

Over-expression of the *ccn2* transgene in chondrocytes of the transgenic mice was confirmed by Northern and Western blot analyses. Northern blot hybridization of total RNA extracted from rib cage chondrocytes of E18.5 embryos with probes for *LacZ* and *ccn2* showed a reaction with the same 6-kb transcript in transgenic, but not wt, chondrocyte RNA (Fig. 1E, *LacZ* and *ccn2*). The intensity of the transgene signal obtained with the *ccn2* probe was about 75% of that of the endogenous *ccn2* mRNA (Fig. 1E, middle panel). Endogenous *ccn2* mRNA was also up-regulated (∼110% of wild type) in transgenic cartilage (Fig. 1E, *ccn2*), possibly due to an autocrine mechanism. The HA-tagged CCN2 protein was detected in cell lysates from transgenic rib cartilage by Western blot analysis using an anti-HA antibody (Fig. 1F).

**Figure 2:**
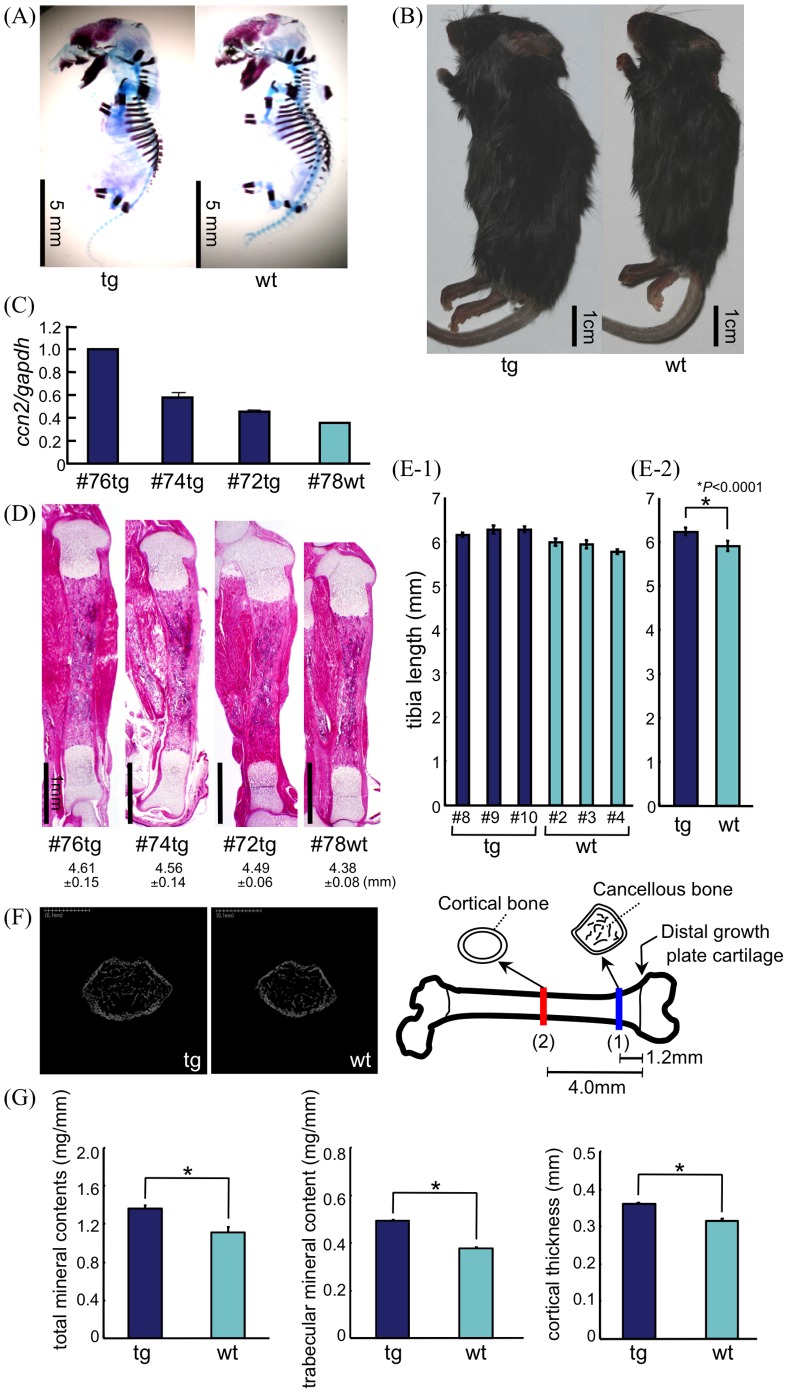
Skeletal analysis of *Col2a1-ccn2* transgenic mice. (A) Skeletal preparation of representative tg and wt littermates at E15.5 after alizarin red and Alcian blue staining. Skeletal development in tg mice appeared normal at this stage. (B) At 8 weeks the transgenic mice consistently showed an ∼12% increase in body size. (C) Quantitative analysis by real-time PCR of *ccn2* mRNA levels in primary cultures of tg and wt rib chondrocytes revealed high-expressing transgenic mice (e.g., #76,#74) in each litter, besides low-expressing littermates (#tg72), the latter of which expressed *ccn2* at about the same level as the wt littermates (see also Fig. 2D). Real time-RCR analysis was repeated at least 2 times for each RNA preparation, and the 2 founder lines showed similar variations, but basically the same results. (D) Hematoxylin-eosin (HE) staining of transgenic and wild-type P1 tibiae from the same littermates as shown in Fig. 2C. Tibiae from transgenic mice showed a relatively extended length of the diaphyses in the high-expressing transgenic littermates. Tg and wt with a number indicate transgenic and non-transgenic littermates, respectively. Six litters from 2 different founder lines were investigated. (E-1) Diaphysis length of tibiae from transgenic and wild-type littermates of a P3 litter. Tibial diaphysis lengths of only pups that showed significantly enhanced levels of *ccn2* mRNA, measured in primary cultures of rib chondrocytes were measured. Serial sections (5–7 slides) were randomly selected every 3 slides from a single tibia, and stained with HE. The images were incorporated into a computer, and the length of diaphyses were measured. Bars indicate the mean length and standard deviations of diaphyses of tibia from wild-type and transgenic littermates (e.g. 2 wt, 8 tg). (E-2) Mean length of diaphyses of tibiae from the wild-type and transgenic mice indicated in E-1. *: p<0.0001. Two different founder lines with 3 litters each were analyzed and similar results were obtained. (F) Left: Representative micro-CT image (cross section) of femora of 8-week-old tg and wt littermates. Right: Positions of measurement in femur. (G) Peripheral quantitative computed tomography analysis of bone density and mineral content was made at 2 sites, one 1.2 mm (site #1, blue), and the other 4.0 mm (site #2, red), distal to the growth plate, as indicated in “F” (right). Bars represent the mean ±SD (n = 9, males). In transgenic bones significant enhancement was seen in total mineral content (tg: 1.36±0.08 mg/mm *vs.* wt: 1.10±0.12 mg/mm), in trabecular mineral content (tg: 0.49±0.01 mg/mm *vs.* wt: 0.38±0.01 mg/mm), and in cortical thickness (tg: 0.060±0.013 mm *vs.* wt: 0.049±0.021 mm); but only in the femora at site #1 were the differences significant (*P<0.05).

At day E15.5 of embryonic development, no major abnormalities in cartilage or bone development were detected in the transgenic animals (Fig. 2A). At 8 weeks, however, the majority of the transgenic mice were about 12% larger than their wild-type littermates (Fig. 2B).

For detailed analysis of the morphological alterations in the skeleton of postnatal transgenic mice, tibiae of transgene and wild-type newborns were sectioned, and their length was measured. The levels of *ccn2* mRNA in chondrocytes cultured from rib cartilage of the same animal were also monitored. Quantitative real-time PCR analysis of *ccn2* mRNA levels in rib chondrocytes in primary culture revealed high-expressing transgenic mice (e.g., #76,#74), as well as low-expressing transgenic littermates (#tg72) in the same litter, which expressed *ccn2* at about the same level as the wt littermates (Fig. 2C). Comparison of tibial length and the *ccn2* mRNA expression level of chondrocytes prepared from rib cartilage of the same animal showed a positive correlation (Fig. 2C and D). The length of diaphyses of tibiae from wt and transgenic littermates at the P3 stage was also measured. The expression level of *ccn2* mRNA in primary cultures of rib chondrocytes from littermates was monitored, and tibiae from pups with significantly higher levels of *ccn2* mRNA compared with wt levels were used for comparison of length of diaphyses (Fig. 2E–1). Between 5–7 slides were randomly selected from serial sections of each tibia and stained with HE; and the length of diaphyses was measured by using an image analysis program. All of the transgenic tibiae with significantly enhanced expression levels of *ccn2* mRNA showed increased tibial length as compared with the wt tibiae (Fig. 2E–1). Comparison of mean length of diaphyses from 3 wt (5.897±0.116 mm) and 3 transgenic mice (6.225±0.080 mm) showed a significant difference (P<0.0001, Fig. 2E–2).

### Over-expression of CCN2 Increased Bone Density, Extent of Mineralization of Cancellous Bone, and Thickness of Cortical Bone

Further evidence for a stimulation of bone growth by CCN2 in transgenic animals was obtained when bone density and mineral content of cancellous bone were monitored by using peripheral-quantitative computed-tomography (pQCT) analysis. For these studies, 8-week-old femora from 4 wt and 5 tg littermates were analyzed for mineral content and cortical bone thickness at 2 sites, one 1.2 mm, and the other more central 4.0 mm distal from the growth plate (Fig. 2F). Significant differences (p<0.05) in total mineral content (tg: 1.36±0.08 mg/mm *vs.* wt: 1.10±0.12 mg/mm), trabecular mineral content (tg: 0.49±0.01 mg/mm *vs.* wt: 0.38±0.01 mg/mm), and cortical thickness (tg: 0.060±0.013 mm *vs.* wt: 0.049±0.021 mm) were observed for the part of the femora closer to the growth plate (Fig. 2F and 2G), but not for the central site (data not shown).

### Over-expression of CCN2 in Chondrocytes Caused Enhanced Accumulation of Extracellular Matrix and Shortened Hypertrophic Zones

**Figure 3:**
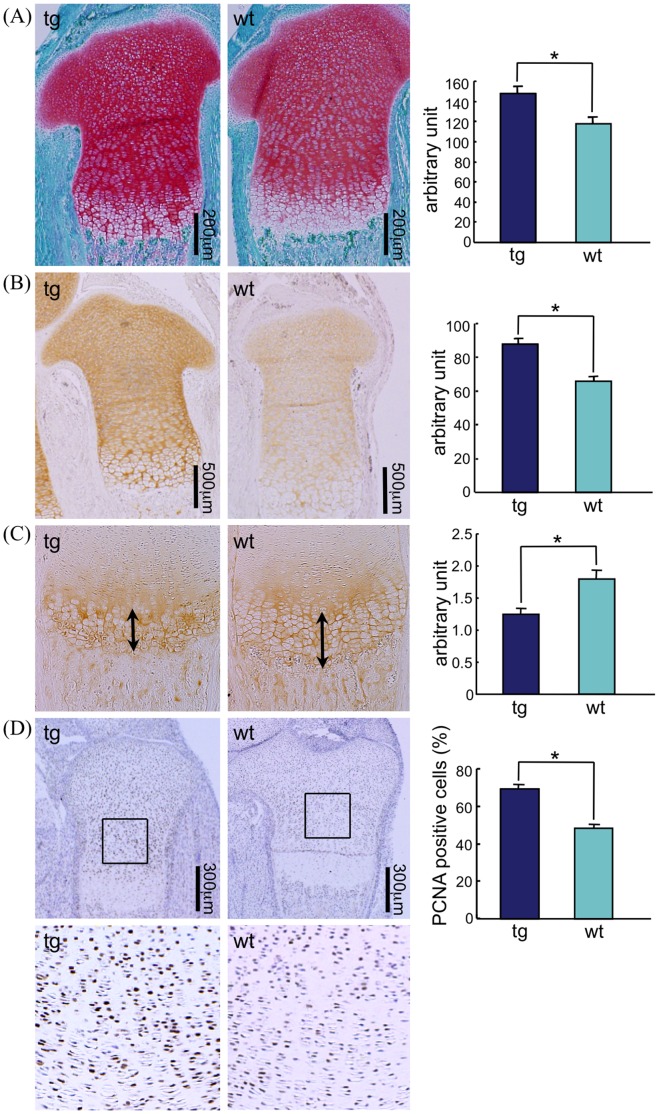
CCN2 overexpression causes enhanced type II collagen and proteoglycan deposition, enhanced chondrocyte proliferation and shortening of the hypertrophic cartilage zone. Tibiae from P1 littermates were stained with safranin-O for proteoglycans (A, left) and with anti-type II collagen antibody (B, left). Whole littermates were analyzed and the color intensity of 3 different wt or tg individuals was measured densitometrically; and the mean values are presented. (A, right; and B, right). *: p<0.005. Typical images from tg and wt littermates are shown. (C, left) Comparison of hypertrophic cartilage zone of CCN2 transgenic littermates. Tibiae were stained with type X collagen antibody. (C, right) The hypertrophic zone of tg cartilage appeared shorter compared with that of the wt cartilage. (D) Immunohistochemical analysis of proliferative cell nuclear antigen (PCNA) in tibiae of *ccn2* tg embryos at E19.5. Proliferative cells were observed in the whole epiphyseal cartilage of tg animals, whereas they were restricted to the proliferative zone of the wt littermates. The number of PCNA-positive cells inside of the boxed area was counted in 5 fields of 3 comparable wt and tg sections. Mean values indicate enhanced chondrocyte proliferation in the tg cartilage (graph at the lower right). *: p<0. 05.

To examine the possibility that the extended skeletal growth of *ccn2* transgenic mice may have been due to enhanced production of cartilage matrix in the epiphysis, we analyzed the extracellular deposition of proteoglycans and type II collagen in the cartilage matrices by staining with safranin O and anti-type II collagen, respectively. Safranin-O staining indicated consistently an enhanced density of proteoglycans in the transgenic cartilage in comparison with cartilage of wt littermates (Fig. 3A). This observation is in accordance with our previous studies showing that CCN2 promotes proteoglycan synthesis in chondrocytes [11]. Also, the immunohistological analysis of type II collagen showed an enhanced reaction in resting chondrocytes and in the growth plate (Fig. 3B and Figure S1A). These results indicate that the over-expression of CCN2 enhanced the production and deposition of extracellular proteoglycans and type II collagen, which is in line with our previous *in vitro* findings. Surprisingly, however, the enhanced matrix deposition did not result in an increase in the size of the cartilaginous epiphysis; rather, the extended bone length was the result of an elongated bony shaft of the diaphysis.

Staining of the skeleton of transgenic embryos with type X collagen antibodies indicated that the hypertrophic zone was shorter in the transgenic embryos than in their wt littermates (Fig. 3C). This observation suggests an acceleration of chondrocyte proliferation and maturation, but possibly also accelerated cartilage resorption and chondrocyte apoptosis in these transgenic animals. Therefore, we next measured chondrocyte proliferation and apoptosis rates in the growing long bones of *ccn2* transgenic animals and their wt littermates.

### Over-expression of CCN2 Resulted in Enhanced Cell Proliferation and Slightly Elevated Apoptosis of Epiphyseal Chondrocytes

In order to assess whether the enhanced bone growth of CCN2 transgenic animals was due to enhanced cell proliferation, we stained sections of E19.5-day transgenic and wt embryos with an antibody against proliferative cell nuclear antigen (PCNA). The data show that over-expression of CCN2 stimulated chondrocyte proliferation predominantly in the proliferative zone, but also in the resting zones (Fig. 3D). This observation is in accordance with previous *in vitro* studies showing that CCN2 promotes chondrocyte proliferation [11].

Curiously, however, staining for apoptotic cells in the growth plate of P3 by using the TUNEL assay revealed slightly, but not significantly, enhanced accumulation of apoptotic cells at the cartilage-bone interface and in the adjacent subchondral zone in the transgenic embryos as compared with their numbers in the wild-type (Fig. S1B). The length of the cartilaginous epiphyses seemed unaffected, since chondrocyte proliferation, cartilage matrix deposition, maturation, cartilage resorption, apoptosis, and assembly of trabecular bone were accelerated by the over-expressed CCN2.

### Over-expression of CCN2 in Chondrocytes Resulted in Enhanced Gene Expression of *Col2a1* and *aggrecan*, and in Enhanced Chondrocyte Maturation *in vitro*

**Figure 4:**
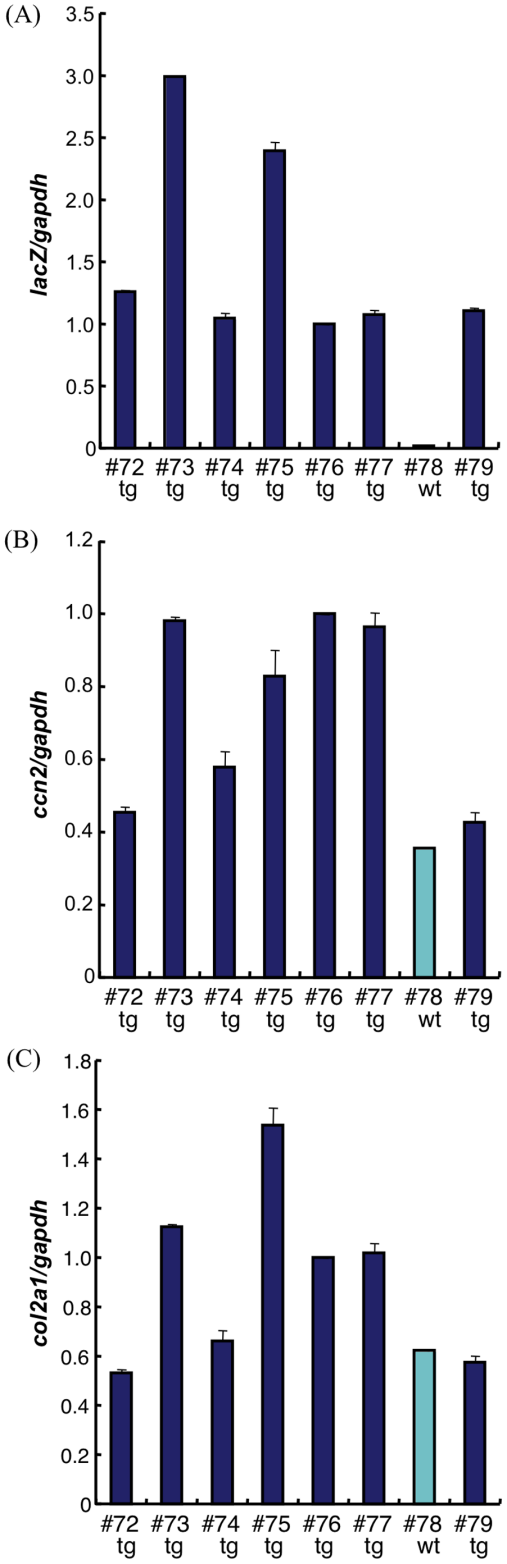
Gene expression analysis reveals enhanced *Col2a1* and *ccn2* in chondrocyte primary cultures of *Col2a1-ccn2* transgenic mice. To obtain high *ccn2* transgene-expressing littermates, we crossed transgenic male and female mice within same founder line; and expression of *LacZ*, *ccn2*, and *Col2a1* mRNA was measured by real-time PCR from 5 d chondrocyte cultures prepared from E18.5 wt and tg embryos. LacZ analysis revealed that high and low *lacZ-*expressing tg littermates and 1 wt were obtained (A). On average, *ccn2* expression levels in tg chondrocytes were significantly higher than those in wild-type littermates (B). *Col2a1* mRNA levels in tg chondrocytes were 2–3 fold higher than those in wt chondrocytes (C). Primary cultures of rib chondrocytes from individual littermates were prepared 3 times from each of the 2 founder lines, and total RNA were prepared. Real time-RCR analysis was repeated at least 2 times for each RNA preparation; and the 2 founder lines showed similar variations, but gave basically the same results. Primary-chondrocytes from *ccn2* tg and wt littermates were also pooled; and gene expression was analyzed as shown in figure S2.

The increased accumulation of proteoglycan and type II collagen in the cartilage matrix of transgenic animals raised the question as to whether *ccn2* over-expression in chondrocytes stimulated cartilage and bone growth by enhancing cell proliferation, by stimulating the production of extracellular matrix or by accelerating the differentiation and maturation of chondrocytes. To obtain high *ccn2* transgene-expression, we crossed transgenic male and female mice and monitored the effects of over-expression of CCN2 in chondrocytes on the expression of extracellular matrix genes. RNA was extracted from short-term primary cultures of rib-cage chondrocytes from E18.5 transgenic or wild-type embryos and analyzed for *lacZ, ccn2*, and *Col2a1* mRNA levels by quantitative real-time PCR. The data showed about equal levels of lacZ expression in chondrocytes of transgenes #72,74, 76, 77 and 79, and a 2–3 fold higher level of the *lacZ* expression in tg #73 and #75, indicating that offspring #73 and #75 may bear double copies of transgene (Fig. 4A). Accordingly, the *ccn2* level in chondrocytes derived from those embryos (#73, 75 tg) was 2–3 fold enhanced as compared with the level for the wt chondrocytes (#78 wt, Fig. 4B). Also tg #76 and #77 showed enhanced levels of *ccn2* expression, whereas *ccn2* expression levels in tg #72, #74 and # 79 were not much higher than endogenous *ccn2* levels measured in the wt embryo #78, perhaps due to inactivation of the transgene. Tg chondrocytes with high over-expression of *ccn2* mRNA (#73, 75 tg), but also tg chondrocytes of #76 and 77 showed enhanced levels of *Col2a1* mRNA as compared with the wt level (#78 wt), as revealed by real-time PCR analysis (Fig. 4B and C), whereas tg cultures with low overexpression of *ccn2* (#72, 74, and 79) showed also low *col2a1* expression. To confirm the enhanced expression of *Col2a1* as well to estimate that of *aggrecan* mRNA in tg chondrocytes, we pooled primary–cultured chondrocytes from tg and wt littermates, and determined their *ccn2, col2a1, aggrecan* mRNA levels (figure S2). The levels of all 3 mRNA were greater in the tg than in the wt pooled cells.

**Figure 5:**
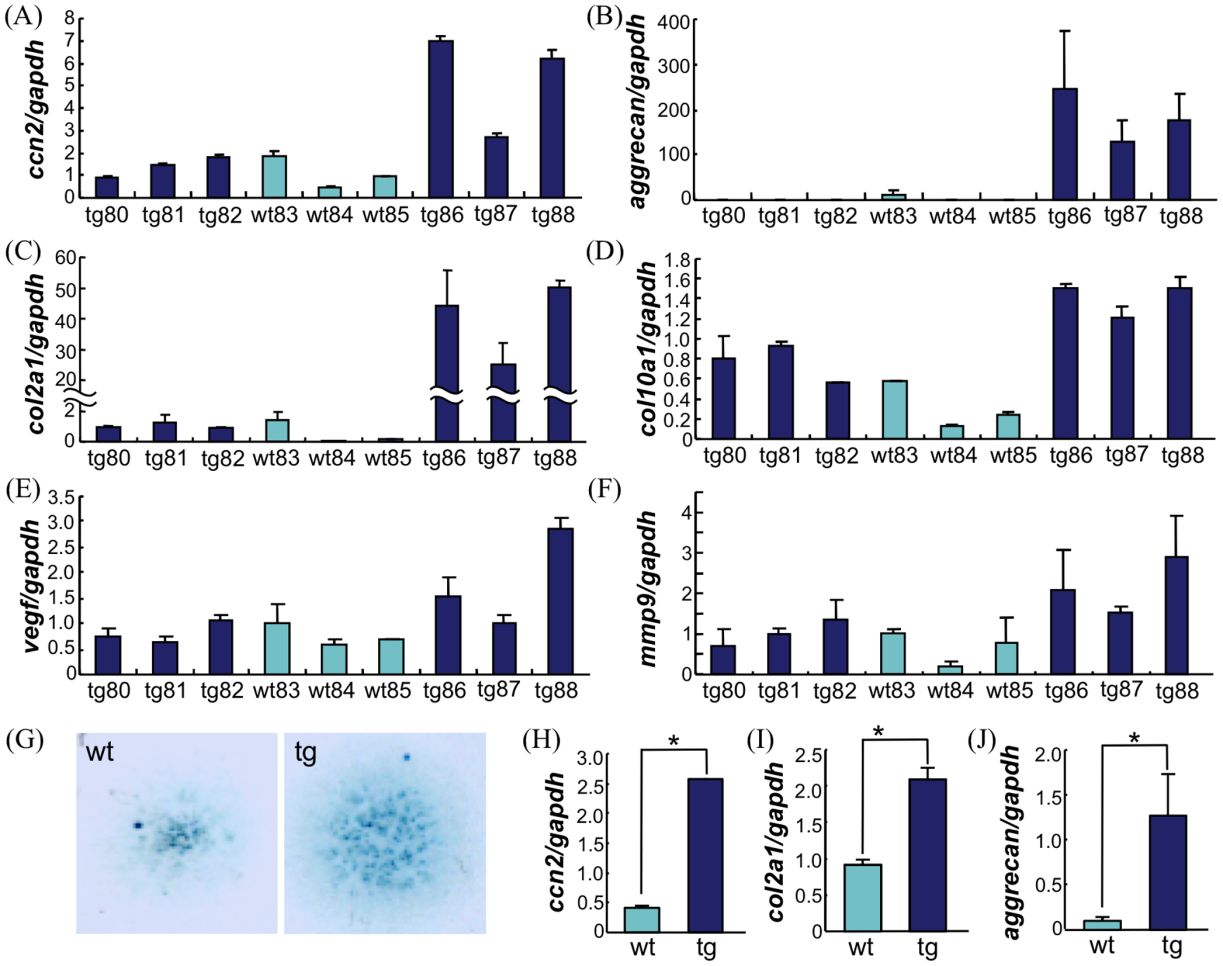
*Ccn2* overexpression on *Col2a1-ccn2* transgenic mice stimulates expression of marker gene of late hypertrophy and chondrogenesis of limb bud mesenchymal cells. For real-time PCR analysis of gene expression, primary chondrocytes isolated from ribs of *ccn2* tg and wt littermates were cultured for 1 month under differentiation-promoting conditions (A–F). In high-expressing tg samples, high levels of *ccn2* mRNA were retained during the entire culture time; whereas low expressers showed *ccn2* mRNA levels similar to those of wt chondrocytes (A). Expression of ECM components such as *aggrecan* (B) and *Col2a1* (C) was strongly up-regulated in the cultures that high levels of *ccn2* mRNA. Markers of late hypertrophic chondrocytes such as *Col10a1* (D), *vegf* (E), and *mmp9* (F) *were* also upregulated in those cultures. Expression levels of 1 representative litter out of 3 litters are shown. Primary 1 month cultures of rib chondrocytes from individual littermates were prepared twice from 2 founder lines; and total RNA was extracted. Real time-RCR analysis was repeated at least twice for the each RNA preparation. The 2 founder lines showed similar variations, but basically the same results. (G–J) Micromass cultures of mesenchymal cells derived from tg and wt E11.5 littermates. After 3 days in culture, nodule formation was accelerated in the cultures derived from ccn2-overexpressing mice as shown by Alcian blue (pH 1, G) and RNA was extracted for real-time PCR analysis. Cultures prepared from *ccn2* over-expressing mice showed enhanced expression of *ccn2* (H), *Col2a1* (I), and *aggrecan* (J) mRNA. *: p<0.005. RNA of each littermates was individually analyzed and nodule formation among *ccn2* wt or tg was similar. Typical images of *ccn2* wt and tg are shown. Real time-RCR analysis was repeated at least 2 times for each RNA preparation. Micromass cultures of mesenchymal cells derived from E11.5 littermates were prepared 4 times from 2 founder lines, and basically similar results were obtained.

The enhanced levels of *Col2a1* mRNA in the transgenic chondrocytes were also retained after 1 month in culture. During that time, chondrocytes ceased to proliferate and started to mature, but the *ccn2* transgene over-expression in tg cultures #86–88 remained at a high level compared with wt cultures #83–85 or low expressing tg cultures #80–82 (Fig. 5A). Primary cultures of chondrocytes with high levels of over-expressed *ccn2* mRNA continued to show strongly elevated *aggrecan* (Fig. 5B, 15-20,000 fold enhancement) and *Col2a1* (Fig. 5C, 100–1000 times enhancement) mRNA levels. The expression of *Col10a1*, a marker of hypertrophy, and that of *vegf* and of *mmp-9*, both vascular invasion factors expressed in the hypertrophic zone and boundary between cartilage and bone, were also enhanced; but not at the same extent as the enhancement of aggrecan and *col2a1* expression (Fig. 5D, 3–10 fold; 5E, 1.5–3 fold; and 5F, 1.5–3 fold enhancement). These results are in accordance with *in vitro* studies on the effect of *ccn2* on cultured chondrocytes [11] and are consistent with the notion that *ccn2* over-expression stimulated chondrocyte maturation.

### Over-expression of *ccn2* Under the Control of the *col2a1* Promoter Accelerated Chondrogenesis

To investigate the effect of over-expression of *ccn2* on chondrogenic differentiation, we prepared micromass cultures of mesenchymal cells from 11.5-day embryonic transgenic and wt mouse limb buds. Mesenchymal cells from transgenic embryos started to develop Alcian blue-positive cartilaginous nodules after 2 days in culture (data not shown). After 3 days the cartilaginous nodule formation was significantly enhanced in cultures prepared from *ccn2*-overexpressing limb-buds cells as compared with that wild-type cells (Fig. 5G). The gene expression of *ccn2, Col2a1* and *aggrecan* was also up-regulated in *ccn2* transgenic micromass cultures as measured by quantitative RT-PCR (Fig. 5H, I and J, respectively).

### Over-expression of CCN2 Enhanced Expression of *IGF-I* and *IGF-II*

**Figure 6:**
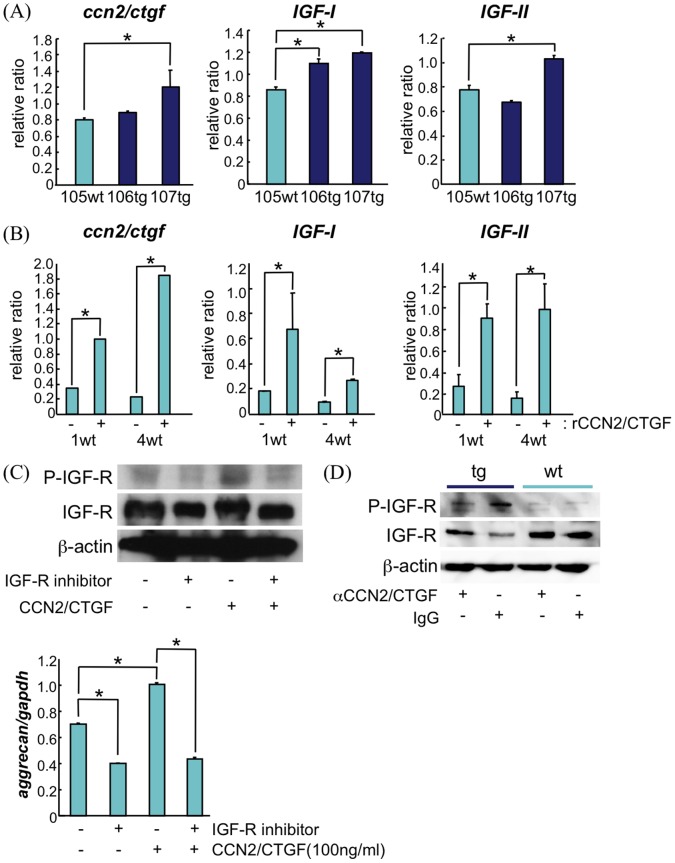
CCN2 stimulates IGF-IGFR pathway. Enhanced expression of IGF-I and IGF-II in primary cultures was found in primary cultures of chondrocytes prepared from the cartilage of *ccn2*-over-expressing mice, and in wt chondrocytes after treatment with recombinant CCN2. (A) Real-time PCR analysis of total RNA from tg cartilage which showed higher expression of *ccn2 (107* *tg)* than wt cartilage (105 wt) also showed enhanced expression of *IGF-I* and *II,* whereas 106 tg with low *ccn2* overexpression showed no enhanced *IGF-II*, but enhanced *IGF-I* expression. *:p<0.05. (B) Addition of recombinant CCN2 (50 ng/ml) to primary cultures of wt mouse rib chondrocytes stimulated *IGF-I* and *II* mRNA as well as *ccn2* mRNA expression. Primary cultures of chondrocytes were prepared from wt E18.5 embryos; and the cells were seeded at 2×10^5^ cells in 3.5-cm dishes with or without rCCN2 in the media, and incubated for 5 days. mRNA levels were standardized with *gapdh*; and all reactions were done in triplicate. Values for 1 wt and 4 wt are from 2 independently generated cultures. *:p<0.005. (C) Phosphorylation of IGF receptor induced by addition of rCCN2 (100 ng/ml) for 24 hours to primary cultures of wt rib chondrocytes, and inhibition of this phosphorylation of IGFR by PPP, an inhibitor of autophosphorylation of IGFR (upper panel). *Aggrecan* mRNA levels were measured (lower panel) and standardized to *gapdh*; and all reactions were done in triplicate. (D) Enhanced phosphorylation of IGFR in *ccn2-*overexpressing chondrocytes and inhibition of phosphorylation of IGFR by CCN2 antibody. Primary cultures of chondrocytes were pooled from P3 rib cages of *ccn2* tg and wt littermates; and cells were seeded at 2×10^5^ cells in 3.5-cm dishes and cultured for 2 days until the cells had reached the confluent state. CCN2 antibody or control IgG was added to the media, and the cultures were then incubated for 24 hours, after which the cells were collected with lysis buffer.

In order to elucidate the mechanism of growth stimulation by the over-expressed CCN2, we analyzed changes in expression levels of growth factors known to be involved in skeletal growth. Remarkably, the RNA from tg chondrocytes contained clearly enhanced expression levels of *IGF-I* and *IGF-II* mRNA (Fig. 6A). This finding was confirmed by examining primary-cultured chondrocytes pooled from tg and wt littermates (Figure S3). This finding suggests that, in addition to the possible direct effects of over-expressed CCN2, these enhanced levels of *IGF-I* or *II* might have been responsible for the stimulation of cortical bone growth, as well as for the enhanced *Col2a1* and *aggrecan* expression observed in the CCN2-over-expressing mice. To confirm this notion, we treated primary cultures of chondrocytes from 18.5-day wt embryos for 5 days with recombinant CCN2. The result showed a several-fold increase in the levels of *IGF-I* and *IGF-II* mRNA as well as a strong increase in endogenous *ccn2* expression (Fig. 6B). In order to elucidate whether CCN2 stimulated IGF-IGF receptor pathway, we examined the autophosphorylation of the IGF-1 receptor in response to the addition of CCN2. CCN2 enhanced the autophosphorylation of IGF-1 receptor (Fig. 6C and Figure S3), and the addition of PPP, IGFR inhibitor, abolished it (Fig. 6C). The CCN2-enhanced expression of *aggrecan* mRNA was also abolished by the addition of the IGFR inhibitor (Fig. 6C). The *ccn2*-overexpressing chondrocytes from *ccn2* tg rib cartilage showed enhanced phosphorylation of IGFR compared with wt chondocytes; accordingly, the CCN2 neutralizing antibody, 11H3, repressed this autophosphorylation (Fig. 6D). The addition of 11H3 antibody down-regulated the expression of *ccn2, igf1*, and *igf2* mRNA (Figure S4). This finding of enhanced expression of IGF-I and -II in CCN2-transgenic chondrocytes is consistent with our finding of enhanced cortical bone growth and mineralization (see discussion).

## Discussion

Previous *in vitro* studies on the response of rabbit growth-plate chondrocytes in primary culture and human chondrosarcoma cells HCS-2/8 to CCN2 demonstrated not only a significant stimulation of proliferation, differentiation, and enhanced synthesis of hyaline cartilage matrix components such as type II collagen and aggrecan, but also enhanced expression of hypertrophic cartilage proteins such as type X collagen and alkaline phosphatase [11], [13]. Since CCN2 is expressed by prehypertrophic and hypertrophic chondrocytes, these findings indicate that CCN2 acts both in an autocrine and in a paracrine manner to promote chondrocyte proliferation and differentiation events. Thus, it may regulate cartilage matrix synthesis and turnover leading to endochondral ossification [4], [11], [15].

Here we provide experimental evidence in support of a significant role of CCN2 in cartilage development and endochondral ossification *in vivo* in transgenic mice over-expressing CCN2 driven by the cartilage-specific *Col2a1* promoter. Most remarkably, transgenic mice expressing high levels of transgenic CCN2 had greater bone length as compared with their wt littermates. By 8 weeks, some of the tg littermates had greater body mass (∼12%), possibly caused by a better eating with tough skeleton. This morphological phenotype reflects several enhanced cellular activities observed in the transgenic cartilage: i) Chondrogenic differentiation of limb-bud mesenchymal cells from CCN2 transgenic animals was greatly enhanced as compared with that of their wild-type counterparts. ii) Histological analysis of tg cartilage revealed increased type II collagen and aggrecan deposition in the extracellular cartilage matrix, consistent with our *in vitro* data showing that chondrocytes isolated from transgenic animals had highly elevated levels of *Col2a1* and *aggrecan* mRNA shortly after isolation; iii) In long-term cultures, CCN2 transgenic rib chondrocytes also expressed higher levels of *Col10a1*, *vegf* and *mmp9* mRNA than wt chondrocytes, indicating accelerated maturation to hypertrophic chondrocytes. iv) PCNA staining revealed a significant increase in chondrocyte proliferation in resting and growth-plate cartilage of transgenic animals; and (v) CCN2 over-expression also caused slightly enhanced apoptosis of hypertrophic chondrocytes.

One explanation for these effects of the over-expressed CCN2 may be the enhanced levels of *IGF- I* and *IGF- II* mRNA in the transgenic chondrocytes. IGF-I and –II and IGF-binding proteins are known to be most potent regulators of cartilage and bone growth [31], [32], [33]. IGF-I and –II are well known to stimulate proliferation and proteoglycan synthesis in cultured chondrocytes [32], [33], [34]. Transgenic mice with an *IGF-I* gene under the control of the *metallothionein-I* gene promoter weigh 1.3 times more than their non-transgenic littermates [35]. Furthermore, IGF-II is considered to be a fetal growth factor that promotes skeletal growth in young rats [36], [37]. Therefore, it is likely that a substantial part, if not all, of the observed effects seen in the transgenic cartilage were due to the additional IGF-I and -II induced by the over-expressed CCN2.

These unexpected findings require revision of current views on the molecular mechanism of growth stimulation by CCN2 and may provide an explanation for our previous observations on the stimulation of proteoglycan and DNA synthesis by CCN2 in HCS-2/8 chondrosarcoma cells and rabbit chondrocytes [11], [13]. Previous studies have shown an interaction between module 1 of CCN proteins and IGF, suggesting a regulatory role of CCN proteins on IGFs [38], [39]. The data presented here, however, indicate that the up-regulation of IGF-I and -II by CCN2 in mouse chondrocyte cultures occurred at the transcriptional level. To which extent the stimulation of bone growth in the transgenic animals was caused by the up-regulated IGFs or by IGF-independent actions of CCN2 remains to be elucidated.

Surprisingly, the enhanced IGF-I and IGF-II levels in the CCN2 transgenic animals did not cause significant elongation of cartilaginous tissues, for the cartilaginous epiphyses of long bones were about the same size in transgenic animals and their wt littermates. Rather, the increase in bone length was due to an extended length of the diaphyseal bony part of the long bones. A paracrine stimulation of periosteal bone cells by CCN2 overexpressed by adjacent chondrocytes, or by IGFs induced by CCN2, is plausible in light of several *in vitro* studies showing stimulation of osteoblast proliferation and differentiation and mineralization by CCN2 [4], [16], [40]. A significant effect of over-expressed CCN2 on bone growth was also evident from the enhanced thickness of cortical bone and increased bone mineralization seen in transgenic mice as compared with those found in their wild-type littermates.

The hypertrophic zone was shorter in the transgenic animals, even though the cartilaginous epiphyses of the long bones were about the same size as in the wild-type animals. There are possible explanations for this phenomenon: 1) The enhanced chondrocyte proliferation may have been compensated by the increase in chondrocyte hypertrophy. 2) The level of VEGF, which induces vascular invasion of hypertrophic cartilage, and that of MMP9, which degrades cartilaginous matrices, were enhanced; in addition apoptosis was slightly accelerated in transgenic hypertrophic chondrocytes. On the other hand, VEGF- CCN2 complexes as formed *in vitro* have been shown to be degraded by MMPs [41]; and this may be an internal autoregulatory mechanism controlling CCN2 levels in the growth plate.

The results of our present gain-of-function experiment are for the most part in accordance with the findings of a loss-of-function study on CCN2*-*deficient mice [17], which develop skeletal dysmorphisms such as distorted cartilage and bone elements as a result of impaired chondrocyte proliferation and endochondral ossification. In line with the shortened hypertrophic zone observed in our CCN2 transgenic mice, the hypertrophic zone is extended in CCN2-deficient mice. Interestingly, however, CCN2 deficient mice do not show significant alterations in total bone size. Yet, this is in accordance with the notion that the major enhancing effect on bone growth in our CCN2 transgenic mice may have been caused by enhanced levels of IGFs. Thus, although the study on the CCN2-deficient mice confirmed the important role of CCN2 as a regulator of cartilage remodelling during endochondral ossification, the absence of more severe phenotypic alterations in these mice might have been due to redundant effects of other members of the CCN family [17].

In the CCN2 transgenic mouse lines presented here, the extent of bone elongation, as well as the extent of enhancement of *Col2a1* and *aggrecan* mRNA levels correlated with the extent of CCN2 over-expression in transgenic chondrocytes of both founder lines. Besides high-expressing chondrocytes, also transgenic chondrocytes showing low levels of CCN2 expression comparable to those of wt rib chondrocytes were seen in each litter, even when derived from the same founder. Enhanced bone size as well as reduced length of the hypertrophic zone was only observed in tg mice with high levels of CCN2 expression. This was probably due to unpredictable somatic inactivation of the transgene in some embryos and reflects the limitation of this technique, which relies on a random integration of the transgene into the genome. Our previous CCN2-transgenic mice under the control of the *Col9a1* promoter show dwarfism several months after birth and smaller testes, but not so much difference in body length [42]. The expression pattern and timing of *Col9a1* expression, however, differ to some extent from those of *Col2a1,* which may explain the difference in phenotype.

Elucidation of the exact molecular mechanisms involved in IGF-independent, CCN2-regulated chondrocyte responses is still hampered by the fact that currently no specific cell-surface signalling cellular receptor for CCN2 has been identified so far in chondrogenic or osteogenic cells; instead, CCN2 seems to control cellular events by complex interactions with numerous growth factors such as IGFs, and perhaps through integrins and their signalling pathways [2], [43], [44].

In conclusion, our study demonstrates that the use of the *Col2a1* promoter for specific over-expression of CCN2 or other members of the CCN family in chondrocytes may represent – together with *ccn2-*deficient chondrocytes - a powerful tool to provide further insight into the specific role of these growth factors in cartilage metabolism and skeletal development.

## Supporting Information

Figure S1 **Accumulation of type II collagen and slightly enhanced apoptosis in** ***ccn2*** **-overexpressing epiphyseal cartilage.** (A) Comparison of accumulation of type II collagen in cartilage of *ccn2*-overexpressing and wt mice. Tibiae from P3 littermates were stained with anti-type II collagen antibody. The color intensity was measured densitometrically. Four wt and 5 *ccn2* tg littermates were analyzed. (B) TUNEL assay on tibiae from P3 littermates shows slightly enhanced apoptosis in the cartilage-bone transition zone in the tg mice.
(TIF)

Figure S2 **Gene expression analysis in pooled primary chondrocytes from** ***ccn2*** **tg and wt littermates.** Expression analysis of *ccn2*, *Col2a1*, and *Aggrean* mRNA of primary chondrocytes from pooled *ccn2* tg and wt littermates. Real time-RCR analysis was done in duplicate, *: p<0.005. The experiments were repeated 3 times and showed similar results.
(TIF)

Figure S3 **Phosphorylation analysis of primary-cultured** ***ccn2 t*** **g and wt chondrocytes pooled from different transgenic line from** **figure 6A** **.** Results of Western blot analysis of IGF-1R and phospho-IGF-1R (upper photos) and those of gene expression analysis (graphs at bottom) of the same cells as used in Western blot analysis are shown. Real time-RCR analysis was done in duplicate and repeated 3 times, *: p<0.005.
(TIF)

Figure S4 **Change in gene expression level of** ***ccn2*** **,** ***igf1*** **, and** ***igf2*** **mRNA by the addition of CCN2 antibody (11H3) to primary cultures of mouse rib chondrocytes from P3 littermates of** ***ccn2*** **tg mice.** Cells from these cultures were seeded at 2×10^5^ cells in 3.5-cm dishes and cultured for 2 days until the cells had reached to confluence. CCN2 antibody or control IgG was added to the media. The cells were incubated for 24 hours, and total RNA was then extracted from them. Real-time PCR demonstrated that CCN2 antibody repressed gene expression of *ccn2*, *igf1*, and *igf2* mRNA in the *ccn2*-overexpresssing chondrocytes. Real time-RCR analysis was done in duplicate, *: p<0.005. The experiments were repeated for 3 times and showed similar results.
(TIF)
