# Supplementary figures and images for: Cartilage–Specific Over-Expression of CCN Family Member 2/Connective Tissue Growth Factor (CCN2/CTGF) Stimulates Insulin-Like Growth Factor Expression and Bone Growth
Source: PLoS One. 2013 Mar 28;8(3):e59226. doi: 10.1371/journal.pone.0059226 (PMC3610707; doi:10.1371/journal.pone.0059226)

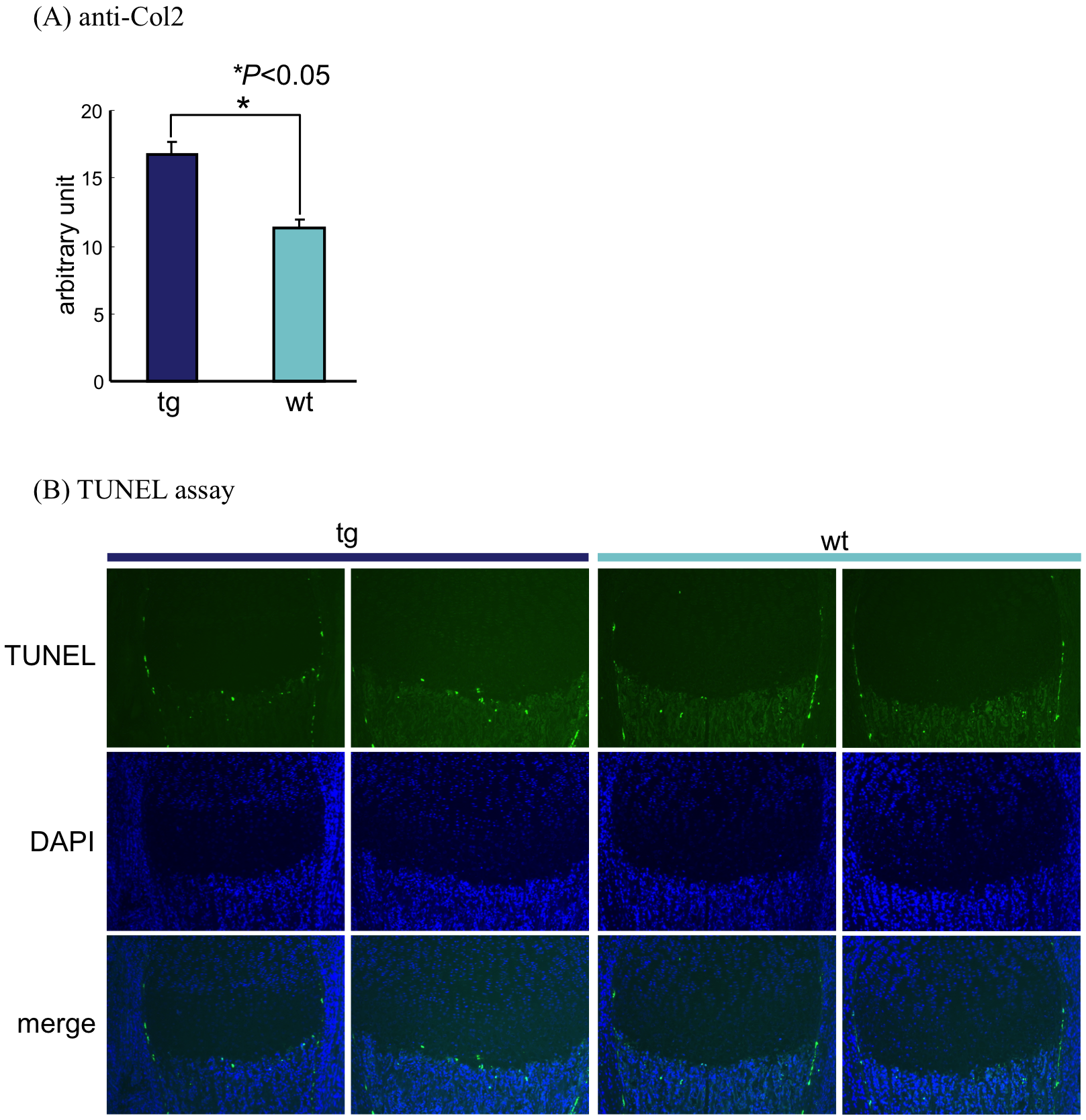

Supplement: Figure S1 — Accumulation of type II collagen and slightly enhanced apoptosis in ccn2 -overexpressing epiphyseal cartilage. (A) Comparison of accumulation of type II collagen in cartilage of ccn2-overexpressing and wt mice. Tibiae from P3 littermates were stained with anti-type II collagen antibody. The color intensity was measured densitometrically. Four wt and 5 ccn2 tg littermates were analyzed. (B) TUNEL assay on tibiae from P3 littermates shows slightly enhanced apoptosis in the cartilage-bone transition zone in the tg mice. (TIF) [file pone.0059226.s001.tif]

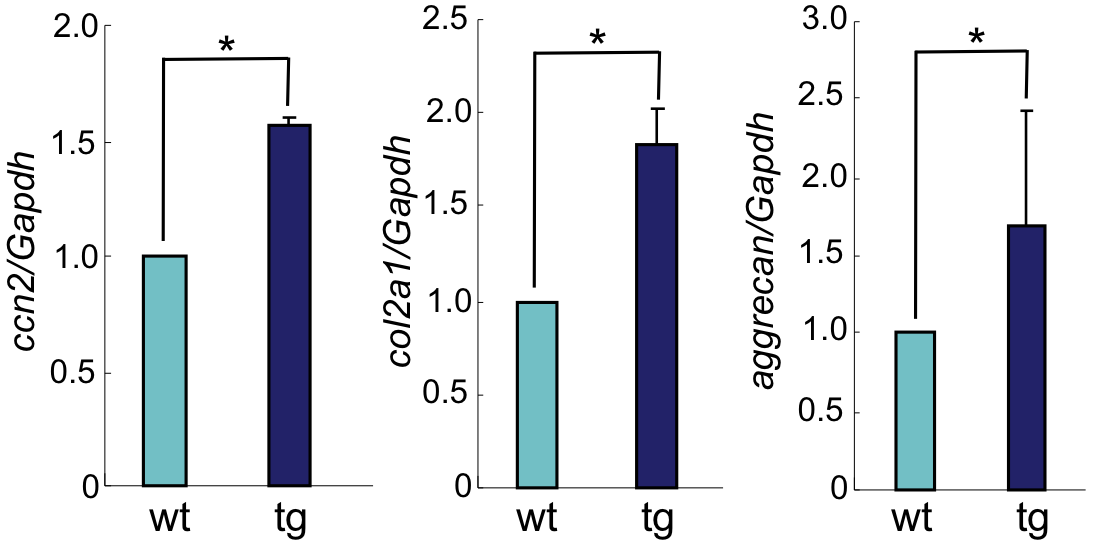

Supplement: Figure S2 — Gene expression analysis in pooled primary chondrocytes from ccn2 tg and wt littermates. Expression analysis of ccn2, Col2a1, and Aggrean mRNA of primary chondrocytes from pooled ccn2 tg and wt littermates. Real time-RCR analysis was done in duplicate, *: p<0.005. The experiments were repeated 3 times and showed similar results. (TIF) [file pone.0059226.s002.tif]

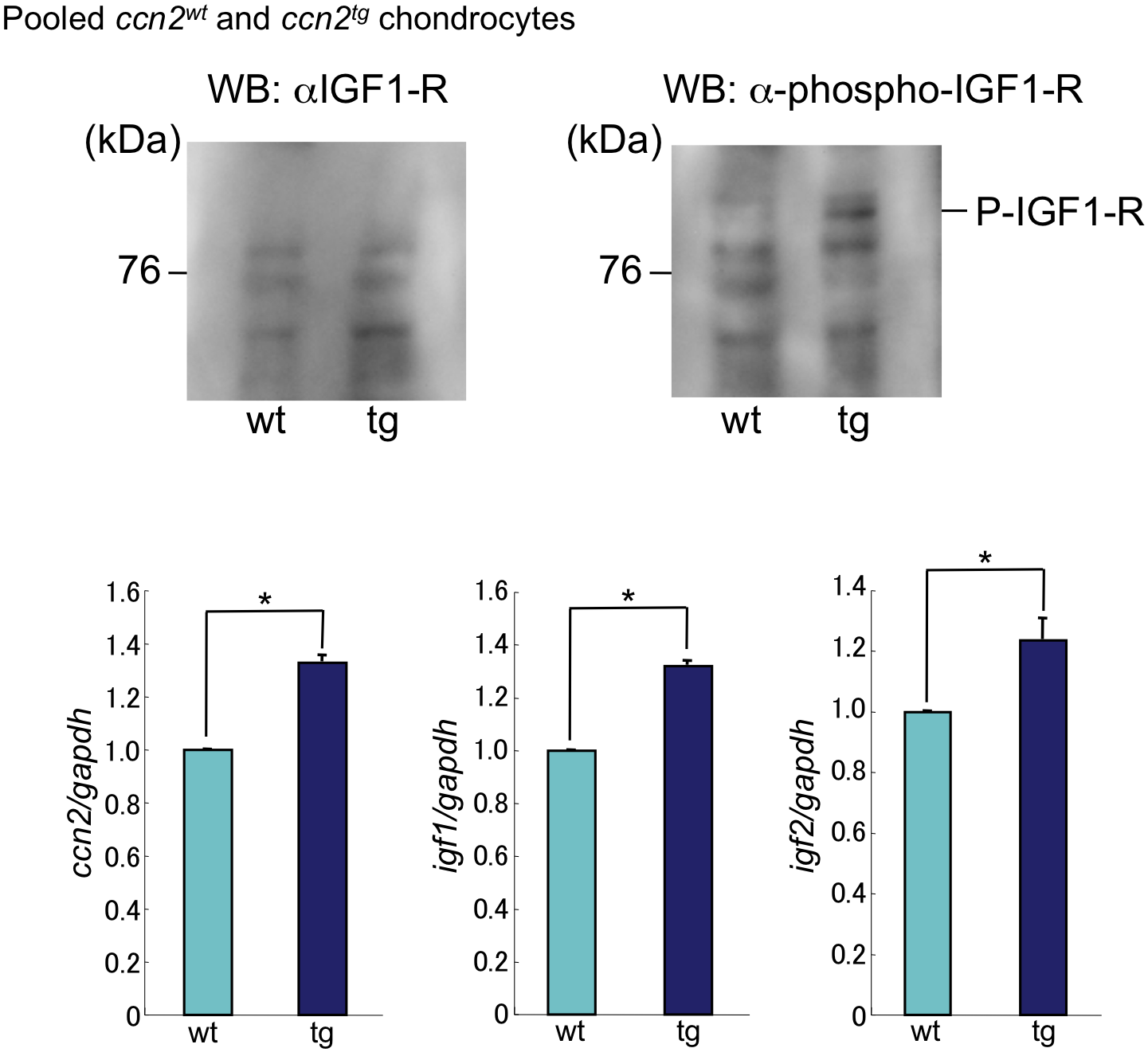

Supplement: Figure S3 — Phosphorylation analysis of primary-cultured ccn2 t g and wt chondrocytes pooled from different transgenic line from figure 6A . Results of Western blot analysis of IGF-1R and phospho-IGF-1R (upper photos) and those of gene expression analysis (graphs at bottom) of the same cells as used in Western blot analysis are shown. Real time-RCR analysis was done in duplicate and repeated 3 times, *: p<0.005. (TIF) [file pone.0059226.s003.tif]

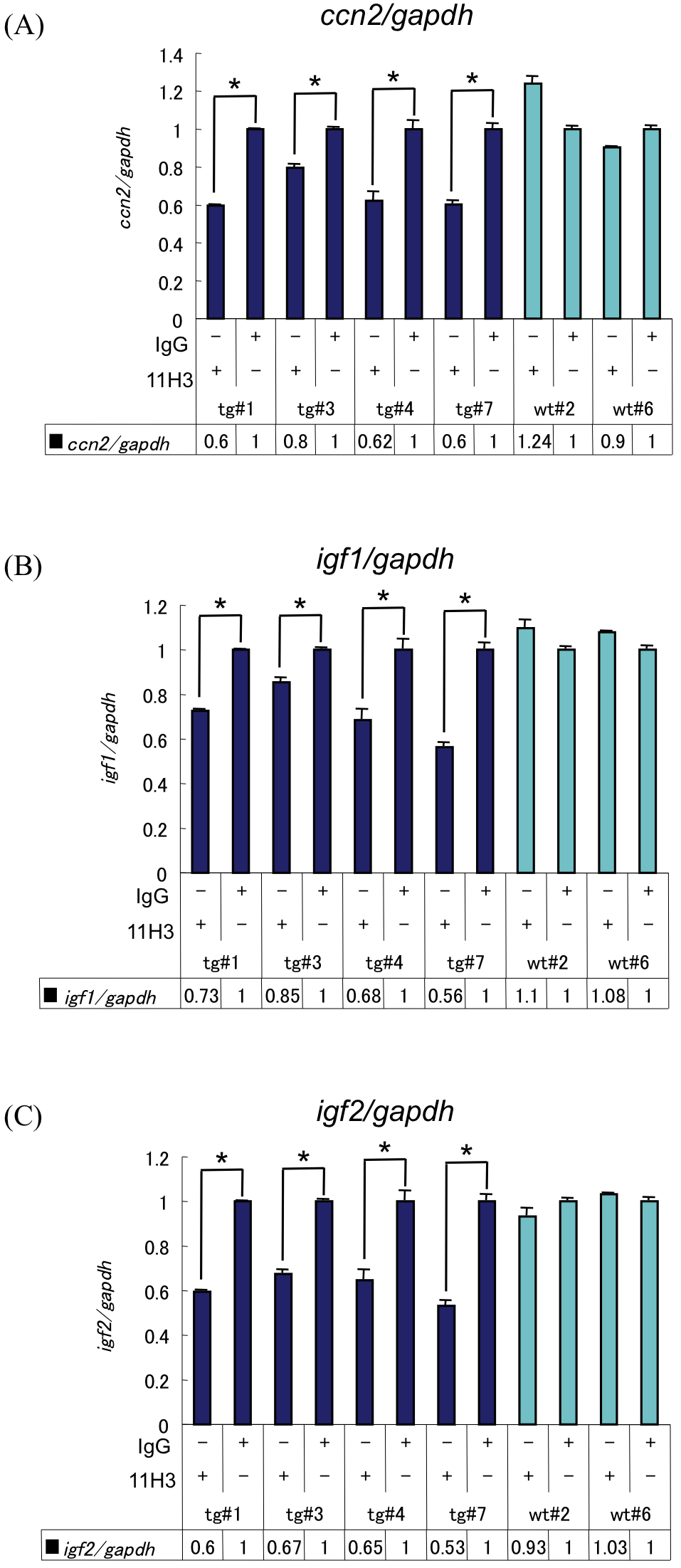

Supplement: Figure S4 — Change in gene expression level of ccn2 , igf1 , and igf2 mRNA by the addition of CCN2 antibody (11H3) to primary cultures of mouse rib chondrocytes from P3 littermates of ccn2 tg mice. Cells from these cultures were seeded at 2×10^5^ cells in 3.5-cm dishes and cultured for 2 days until the cells had reached to confluence. CCN2 antibody or control IgG was added to the media. The cells were incubated for 24 hours, and total RNA was then extracted from them. Real-time PCR demonstrated that CCN2 antibody repressed gene expression of ccn2, igf1, and igf2 mRNA in the ccn2-overexpresssing chondrocytes. Real time-RCR analysis was done in duplicate, *: p<0.005. The experiments were repeated for 3 times and showed similar results. (TIF) [file pone.0059226.s004.tif]
